# Association of fluid balance trajectories and cumulative fluid balance with in-hospital mortality in patients with acute pancreatitis

**DOI:** 10.3389/fmed.2026.1863757

**Published:** 2026-07-29

**Authors:** Fei Wang, Youqing Xu, Xin Li

**Affiliations:** Department of Gastroenterology, Beijing Tiantan Hospital, Capital Medical University, Beijing, China

**Keywords:** acute pancreatitis, fluid accumulation, fluid balance, mortality, trajectory

## Abstract

**Background:**

Fluid resuscitation represents a cornerstone of early management in acute pancreatitis (AP). Accumulating evidence suggests that positive fluid balance (FB) is independently associated with adverse outcomes, including pulmonary edema and abdominal compartment syndrome. This study aimed to identify distinct FB trajectories during the initial 72 h in ICU-admitted patients and comprehensively evaluate their associations with in-hospital mortality, incorporating both dynamic trajectory patterns and static cumulative measures.

**Methods:**

We analyzed data from 1,026 adult AP patients admitted to the ICU using the Medical Information Mart for Intensive Care IV (MIMIC-IV) database. An external validation cohort of 271 patients from the eICU Collaborative Research Database (eICU-CRD) was used for replication. Latent class mixed models (LCMM) were employed to identify distinct FB trajectories. The primary outcome was in-hospital mortality; secondary outcome was ICU length of stay (LOS). Restricted cubic splines (RCS) were used to assess nonlinear associations between cumulative FB (CumFB) and outcomes.

**Results:**

Three distinct FB trajectories were delineated: Low FB (80.02%, consistently low net balance), High-Fall FB (11.99%, initially high volume followed by rapid de-escalation), and Rise-Fall FB (7.99%, progressive early accumulation peaking before declining). The Rise-Fall trajectory was independently associated with higher in-hospital mortality and prolonged ICU LOS. RCS revealed a U-shaped association between CumFB and mortality, with risks rising at CumFB >25 mL/kg—a threshold similarly associated with extended ICU LOS. These findings were corroborated in the eICU-CRD cohort.

**Conclusion:**

The Rise-Fall FB trajectory is independently associated with poor outcomes, manifesting as both increased mortality and prolonged ICU LOS, while CumFB exhibits a nonlinear association with outcomes.

## Introduction

Acute pancreatitis (AP) is a common, potentially life-threatening inflammatory condition whose global incidence is rising rapidly (1–3). It’s clinical course ranges from a self-limited, mild illness to a fulminant systemic syndrome with multi-organ failure. The initiating pancreatic injury provokes the release of inflammatory mediators and increases capillary permeability, leading to rapid extravasation of protein-rich fluid into the third space and consequent relative intravascular depletion, distributive shock and microcirculatory dysfunction (4, 5). Therefore, intravenous fluid resuscitation remains a cornerstone of early AP management, intended to restore intravascular volume, optimize pancreatic microcirculation and prevent organ hypoperfusion (6).

Historically, early aggressive resuscitation strategies were advocated and associated with improved outcomes in some observational studies (7, 8). However, several high-quality randomized controlled trials have shown that excessive fluid administration does not reduce disease severity and instead increases complications such as pancreatic necrosis, pulmonary oedema, and abdominal compartment syndrome (9–11).

In response, contemporary practice has shifted toward more conservative, response-guided and goal-directed individualized fluid strategies (12–14), embracing a dynamic rather than static concept of resuscitation (15). Nevertheless, the optimal fluid management approach remains unclear, highlighting the need for more granular characterization of fluid exposure patterns in patients with severe AP.

Most prior investigations have focused on static metrics within prespecified time windows, such as cumulative fluid volume or infusion rate (8–10). Although informative, these measures may fail to capture the dynamic evolution of fluid therapy during the early critical phase. Fluid balance (FB) is shaped by multiple interacting factors—infused volume, vascular permeability, renal function and haemodynamics—and is continually regulated by renal, vascular and neural mechanisms (16, 17). Consequently, the temporal pattern of fluid accumulation and resolution may reflect distinct pathophysiological phenotypes and confer differing risks. While FB has been linked to outcomes in heterogeneous critically ill populations (18–22), whether reproducible FB trajectory patterns exist among patients with AP and how these trajectories relate to clinical outcomes remain insufficiently explored.

Moreover, the relationship between fluid exposure and prognosis may be non-linear: both excessive positive and excessive negative balances could be harmful, yet few studies in AP have systematically evaluated such non-linear associations (23).

Notably, while most patients with mild AP have a favorable prognosis, those requiring ICU admission often experience a profound systemic inflammatory response and significant third-spacing, placing them at dual risk of hypovolemic shock and fluid overload. Therefore, focusing on this high-risk adult cohort, we conducted a retrospective analysis of fluid input and output during the first 72 h after ICU admission. We systematically characterized the temporal dynamics of net fluid balance and, by integrating cumulative fluid load metrics with trajectory-based modeling, sought to identify fluid phenotypes and risk thresholds with independent prognostic value. These findings ultimately aim to inform early, evidence-based, personalized fluid management in AP.

## Materials and methods

### Data source and study design

We conducted a retrospective cohort study utilizing two large-scale publicly available databases: the Medical Information Mart for Intensive Care (MIMIC-IV) (version 3.1) and the eICU Collaborative Research Database (eICU-CRD) (version 2.0) (24, 25). To ensure independence of observations, only the first ICU admission for AP was included for each patient, and ICU readmissions were excluded. The MIMIC-IV database includes patient data from ICU admissions at Beth Israel Deaconess Medical Center (BIDMC) between 2008 and 2022. Use of the database was approved by the BIDMC Institutional Review Board (IRB), and all personally identifiable information was anonymized. The eICU-CRD cohort originates from hospitals not affiliated with the MIMIC-IV source institution. Given the retrospective design of the present study and the use of fully de-identified data, the requirement for informed consent was waived by the IRB. Researchers requesting access to these datasets are required to complete ethics training, pass relevant examinations, and obtain appropriate authorization. The principal investigator obtained data use approval and certification after signing the data use agreement (No. 68150503). This study adhered to the ethical principles of the Declaration of Helsinki, and all procedures involving human data were conducted in accordance with these guidelines.

We included adult patients (age ≥ 18 years) with a diagnosis of acute pancreatitis identified by International Classification of Diseases (ICD) codes: ICD-9 (577.0) and ICD-10 (K85). Patients who met the following criteria were excluded: (1) missing survival information; (2) ICU stay < 24 h; (3) fewer than three fluid balance measurements within the first 72 h of ICU admission.

### Data processing

Data were collected on the following variables: age, gender, race, heart rate, respiratory rate, temperature, oxyhemoglobin saturation (SpO2), systolic blood pressure (SBP), mean blood pressure (MBP), diastolic blood pressure (DBP), Sequential Organ Failure Assessment (SOFA) score, Charlson Comorbidity Index (CCI), Acute Physiology Score III (APS III), Simplified Acute Physiology Score II (SAPS II), anion gap (AG), white blood cell count (WBC), platelet, hemoglobin, hematocrit, red blood cell distribution width (RDW), blood urea nitrogen (BUN), creatinine, calcium, sodium, chloride, potassium, glucose, total bilirubin (TBil), prothrombin time (PT), alkaline phosphatase (ALP), alanine aminotransferase (ALT), aspartate aminotransferase (AST), and bicarbonate. The presence of the following conditions or treatments was recorded as yes/no: congestive heart failure, chronic pulmonary disease, severe liver disease, diabetes, chronic kidney disease (CKD), malignant cancer, hypertension, acute kidney injury (AKI), ventilation use, which was defined as invasive mechanical ventilation, vasopressor use, diuretic use, octreotide use and renal replacement therapy (RRT).

Missing data for mortality outcomes were excluded from analysis. Clinical variables with more than 15% missing values were excluded from the analysis. For remaining variables, we applied an imputation strategy: single imputation using the median (for continuous) or mode (for categorical) for variables with < 1% missing data, and multiple imputation by chained equations for variables with 1–15% missing data to preserve statistical power and reduce bias (26).

### Outcome

The primary outcome in this study was in-hospital mortality among patients with acute pancreatitis, defined as any death occurring during hospitalization. Secondary outcomes included 30-day, 90-day, and 1-year all-cause mortality, as well as ICU length of stay (LOS).

### Exposure: fluid balance trajectories and cumulative fluid balance

The key exposure FB, calculated for each 12-h interval during the first 72 h after ICU admission using the following formula: FB (mL/kg) = (fluid input − fluid output)/admission body weight (kg). Fluid input included all intravenous infusions administered via drip or bolus, encompassing both crystalloid and colloid solutions, as well as blood products. Enteral and oral intake (including oral diet and enteral tube feeding) were also included. Fluid output comprised urine output, drainage from body cavities, and estimated gastrointestinal losses. To ensure data accuracy and avoid retrospective contamination, we specifically excluded output entries recorded during the intraoperative anesthesia recovery period and any entries flagged as “prior output” (i.e., output occurring before the formal ICU admission charting time).

We applied latent class mixed modeling (LCMM) to the series of 12-hourly FB values to identify distinct subgroups of patients following similar longitudinal FB patterns. LCMM integrates mixed-effects modeling and latent class analysis, enabling detection of heterogeneous trajectory classes while accounting for within-individual correlation over time (27, 28). The optimal number of classes was determined using statistical fit indices, including: (1) Akaike Information Criterion (AIC) and Bayesian Information Criterion (BIC) should be minimized; (2) entropy values > 0.7; (3) the minimum share of each category should not be < 5%; and (4) the average posterior probability for each category should be > 70% (29). Based on these criteria, three FB trajectory classes were identified (Supplementary Table S1, S2).

The cumulative fluid balance (CumFB) during the 0–72-h period after ICU admission was computed as the net fluid balance over this time interval.

### Statistical analysis

Continuous variables were presented as mean ± standard deviation (SD) for normally distributed data or as median with interquartile range (IQR) for non-normally distributed data; group comparisons were made using analysis of variance or the Kruskal–Wallis test based on data distribution. Categorical variables were expressed as numbers with percentages [n (%)] and were compared using the chi-square test or Fisher’s exact test.

We evaluated the impact of both FB trajectories and CumFB on two primary clinical endpoints: in-hospital mortality and ICU l LOS. To ensure consistency, both analyses employed the same multivariable adjustment strategy. Recognizing that baseline illness severity is a key determinant of outcomes and fluid requirements, covariates were rigorously selected and adjusted for across three nested models: Model 1 was unadjusted; Model 2 was adjusted for covariates selected via backward stepwise selection, which included established severity indices—specifically the Acute Physiology Score III (APS III) and Charlson Comorbidity Index (CCI)—alongside other clinically relevant variables; and Model 3 combined Boruta feature selection with least absolute shrinkage and selection operator (LASSO) regression for robust variable screening and modeling (30). For the primary outcome of in-hospital mortality, a Cox proportional hazards regression model was utilized to estimate hazard ratios (HRs) with 95% confidence intervals (CIs). For the secondary outcome of ICU LOS, multivariable linear regression was performed. Results are reported as *β* with 95% CI, representing the absolute difference in days compared with the reference group.

To further mitigate residual confounding, we performed Inverse Probability of Treatment Weighting (IPTW) (31). This approach balanced baseline characteristics—including comorbidities, laboratory indices, and severity scores—across trajectory groups. Crucially, the association between fluid balance trajectories and mortality was re-evaluated within this pseudo-population to verify the robustness of the findings independent of baseline severity.

Furthermore, we conducted two additional sensitivity analyses. First, we repeated the entire analytical pipeline after excluding patients with an ICU length of stay less than 3 days. This ensured that the FB trajectories were derived from a population with sufficient data density and clinical observation time. Second, to address early deaths, we used competing-risks regression treating death before 72 h as the competing event. This allowed direct assessment of FB trajectories on post-72-h mortality while accounting for competing risks.

Beyond regression modeling, we utilized Kaplan–Meier curves and log-rank tests were used for survival visualization. Subgroup analyses and interaction tests were performed for age, gender, CKD, vasopressor use, and ventilation.

Restricted cubic splines (RCS) were used to fit a multivariable Cox proportional hazards model, with four knots placed at the 5th, 35th, 65th, and 95th percentiles of the exposure distribution. A likelihood ratio test was performed to evaluate deviation from linearity (32). Based on the fitted RCS model, CumFB was categorized into three groups. Subsequent multivariable Cox regression estimated adjusted hazard ratios (HRs) for mortality, while multivariable linear regression evaluated the association with ICU LOS.

To validate the generalizability of trajectory patterns and their mortality associations, we established an independent validation cohort from the eICU-CRD using identical inclusion and exclusion criteria. First, to quantify baseline comparability between the two databases, we compared demographic, clinical, and severity characteristics using standardized mean differences (SMD). Subsequently, we replicated the LCMM framework to identify FB trajectory patterns. Finally, in-hospital mortality associations were quantified using multivariable Cox regression adjusting for key prognostic covariates previously selected via stepwise regression and Boruta-LASSO in the derivation cohort. Results are reported as hazard ratios (HRs) with 95% confidence intervals (CIs).

All statistical analyses were performed using R software (version 4.4.3). A two-sided *p* value < 0.05 was considered statistically significant.

## Results

### Patient baseline characteristics

**Figure 1:**
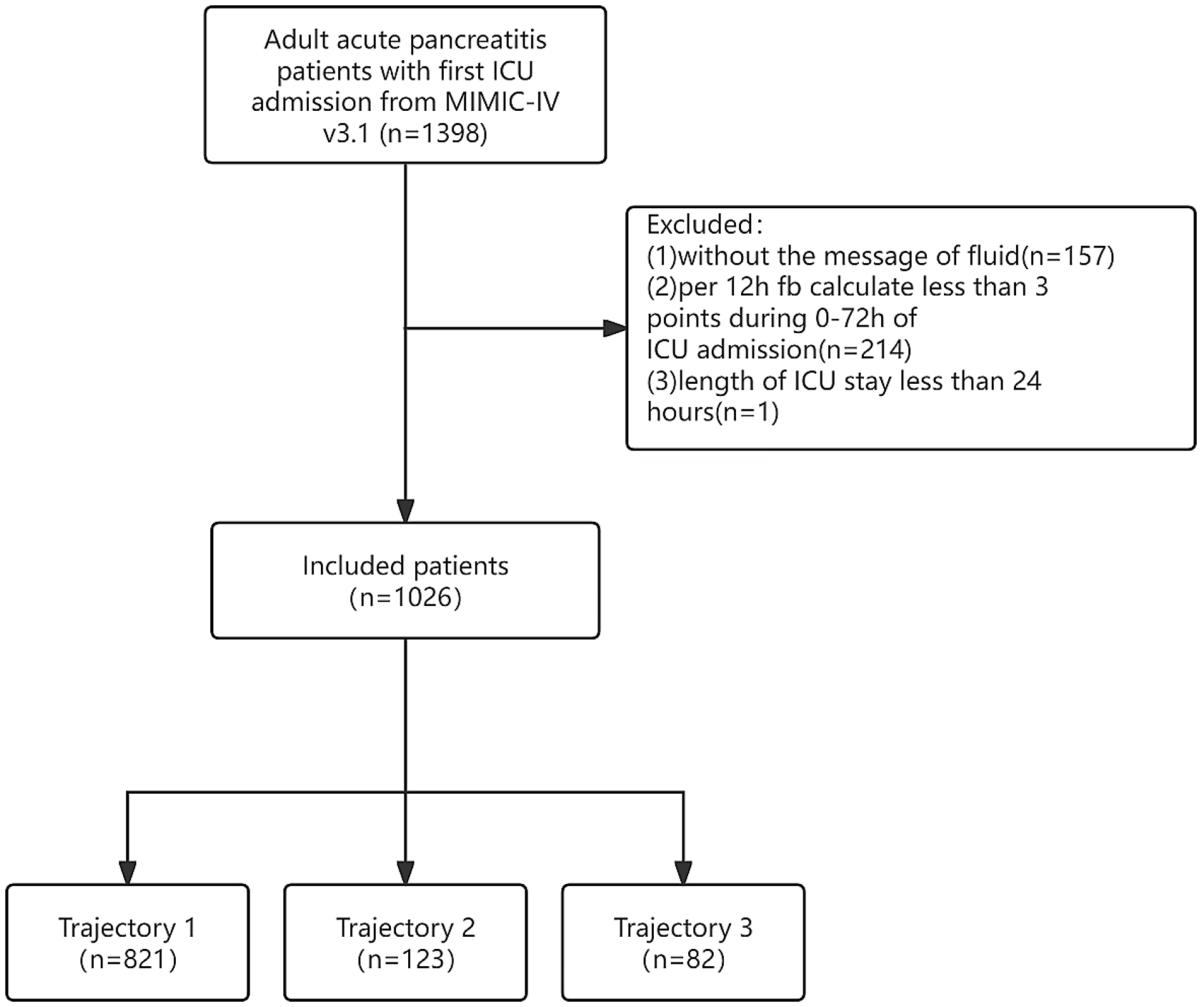
Participant flow diagram illustrating inclusion and exclusion criteria for the MIMIC-IV cohort.

This study extracted 1,398 patients diagnosed with acute pancreatitis and admitted to the ICU for the first time in the MIMIC cohort. After excluding cases with ICU stays shorter than 24 h and missing fluid information, 1,026 patients were ultimately included in the analysis (Figure 1). The median age of the cohort was 58 years, with 41.2% being female, overall in-hospital mortality was 13.5% (Table 1). Table 1 presents the baseline characteristics of population stratified by the identified fluid balance trajectories, showing that use of treatment (RRT, ventilation use, vasopressor use), longer ICU and hospital stays, and higher mortality at in-hospital, 30 days, 90 days, and 1 year were concentrated in the High-Fall FB and especially the Rise-Fall FB trajectories.

**Table 1:** Baseline characteristics according to fluid balance trajectories.

Variables		Overall (*N* = 1,026)	Fluid balance Trajectories			*p*
			Low FB (*n* = 821)	High-Fall FB (*n* = 123)	Rise-Fall FB (*n* = 82)	
Age, years [median (IQR)]		58 [46, 71]	59[46, 72]	54 [44, 68]	55 [46, 69]	0.250
Gender, *n* (%)	Female	423 (41.2)	326 (39.7)	60 (48.8)	37 (45.1)	0.123
	Male	603 (58.8)	495 (60.3)	63 (51.2)	45 (54.9)	
Race, *n* (%)	White	633 (61.7)	517 (63.0)	73 (59.3)	43 (52.4)	0.031
	Asian	38 (3.7)	26 (3.2)	4 (3.3)	8 (9.8)	
	Black	110 (10.7)	87 (10.6)	17 (13.8)	6 (7.3)	
	Other	245 (23.9)	191 (23.3)	29 (23.6)	25 (30.5)	
CumFB, mL/kg [median (IQR)]		28.14 [−3.61, 75.69]	17.54 [−8.87, 46.45]	122.50 [58.75, 208.77]	180.28 [129.51, 240.26]	<0.001
Heart rate, beats/min [median (IQR)]		101 [85, 116]	99[84, 115]	109[90, 125]	105 [90, 120]	0.001
Respiratory rate, breaths/min [median (IQR)]		21 [17, 25]	20[17, 25]	22 [18, 27]	22 [18, 27]	0.003
Temperature, °C [median (IQR)]		36.9 [36.5, 37.3]	36.9 [36.6, 37.3]	36.7 [36.2, 37.2]	36.8 [36.4, 37.4]	0.002
DBP, mmHg [median (IQR)]		72 [60, 87]	73 [61, 88]	65 [55, 83]	71 [58, 84]	0.005
SOFA [median (IQR)]		2 [0, 5]	2[0, 4]	3 [1, 6]	2 [0, 5]	<0.001
CCI [median (IQR)]		3 [2, 6]	3 [2, 6]	4 [2, 6]	4 [2, 6]	0.792
APS III [median (IQR)]		49 [36, 67]	46 [34, 60]	73 [49, 95]	73 [51, 92]	<0.001
SAPSII [median (IQR)]		35 [25, 46]	33 [24, 43]	47 [31, 64]	50 [35, 58]	<0.001
Anion Gap, mmol/L [median (IQR)]		15.00 [13.00, 18.00]	15.00 [13.00, 18.00]	17.00 [13.00, 22.00]	17.00 [13.00, 21.00]	<0.001
WBC, ×10^9^/L [median (IQR)]		12.60 [8.50, 18.10]	12.60 [8.40, 17.90]	11.50 [8.15, 17.60]	14.00 [9.77, 19.15]	0.219
Platelet, ×10^9^/L [median (IQR)]		190.00 [129.00, 275.75]	190.00 [133.00, 276.00]	187.00 [116.00, 277.00]	181.00 [123.50, 264.00]	0.461
Hemoglobin, g/dL [median (IQR)]		10.90 [9.20, 12.50]	10.90 [9.20, 12.50]	11.00 [9.20, 13.35]	11.00 [9.30, 13.10]	0.424
Hematocrit, % [median (IQR)]		32.90 [28.60, 37.80]	32.90 [28.60, 37.50]	32.70 [28.35, 40.00]	33.10 [27.98, 41.53]	0.451
RDW, % [median (IQR)]		14.70 [13.70, 16.30]	14.70 [13.70, 16.30]	14.50 [13.70, 16.10]	15.30 [14.00, 16.82]	0.106
Creatinine, umol/L [median (IQR)]		88.40 [61.88, 159.12]	88.40 [61.88, 141.44]	132.60 [88.40, 238.68]	132.60 [79.56, 274.04]	<0.001
TBil, umol/L [median (IQR)]		17.10 [10.26, 47.88]	17.10 [8.55, 47.88]	17.10 [10.26, 43.61]	21.38 [10.26, 54.72]	0.239
ALT, U/L [median (IQR)]		47.00 [23.00, 129.00]	47.00 [23.00, 127.00]	41.00 [22.00, 130.50]	60.00 [24.00, 213.25]	0.393
Bicarbonate, mmol/L [median (IQR)]		21.00 [18.00, 24.00]	22.00 [19.00, 25.00]	18.00 [13.00, 21.00]	19.00 [16.00, 23.00]	<0.001
CKD, *n* (%)	Yes	187 (18.2)	147 (17.9)	22 (17.9)	18 (22.0)	0.660
Malignant cancer, *n* (%)	Yes	78 (7.6)	64 (7.8)	10 (8.1)	4 (4.9)	0.619
Hypertension, *n* (%)	Yes	461 (44.9)	389 (47.4)	49 (39.8)	23 (28.0)	0.002
AKI, *n* (%)	Yes	565 (55.1)	417 (50.8)	89 (72.4)	59 (72.0)	<0.001
Ventilation use, *n* (%)	Yes	346 (33.7)	240 (29.2)	57 (46.3)	49 (59.8)	<0.001
Vasopressor use, *n* (%)	Yes	328 (32.0)	207 (25.2)	67 (54.5)	54 (65.9)	<0.001
Diuretic use, *n* (%)	Yes	115 (11.2)	96 (11.7)	9 (7.3)	10 (12.2)	0.342
RRT, *n* (%)	Yes	78 (7.6)	31 (3.8)	20 (16.3)	27 (32.9)	<0.001
ICU stay, days [median (IQR)]		3.24 [1.86, 8.18]	2.93 [1.77, 6.04]	5.94 [2.38, 13.89]	9.20 [3.52, 20.87]	<0.001
Hospital stay, days [median (IQR)]		12.82 [6.89, 23.36]	11.71 [6.79, 21.71]	16.73 [7.52, 26.77]	20.69 [10.90, 37.28]	<0.001
In-hospital mortality, *n* (%)	Yes	138 (13.5)	79 (9.6)	29 (23.6)	30 (36.6)	<0.001
30-day mortality, *n* (%)	Yes	130 (12.7)	76 (9.3)	28 (22.8)	26 (31.7)	<0.001
90-day mortality, *n* (%)	Yes	196 (19.1)	129 (15.7)	36 (29.3)	31 (37.8)	<0.001
1-year mortality, *n* (%)	Yes	252 (24.6)	175 (21.3)	39 (31.7)	38 (46.3)	<0.001

Data are presented as median [interquartile range] or number (percentage). IQR, Interquartile Range; CumFB: Cumulative Fluid Balance; DBP: Diastolic Blood Pressure; SOFA: Sequential Organ Failure Assessment; CCI: Charlson Comorbidity Index; APS III: Acute Physiology Score III; SAPS II: Simplified Acute Physiology Score II; WBC: White Blood Cell; RDW: Red Blood Cell Distribution Width; TBil: Total Bilirubin; ALT: Alanine Aminotransferase; CKD: Chronic Kidney Disease; AKI: Acute Kidney Injury; RRT: Renal Replacement Therapy.

### Identification of fluid balance trajectories

**Figure 2:**
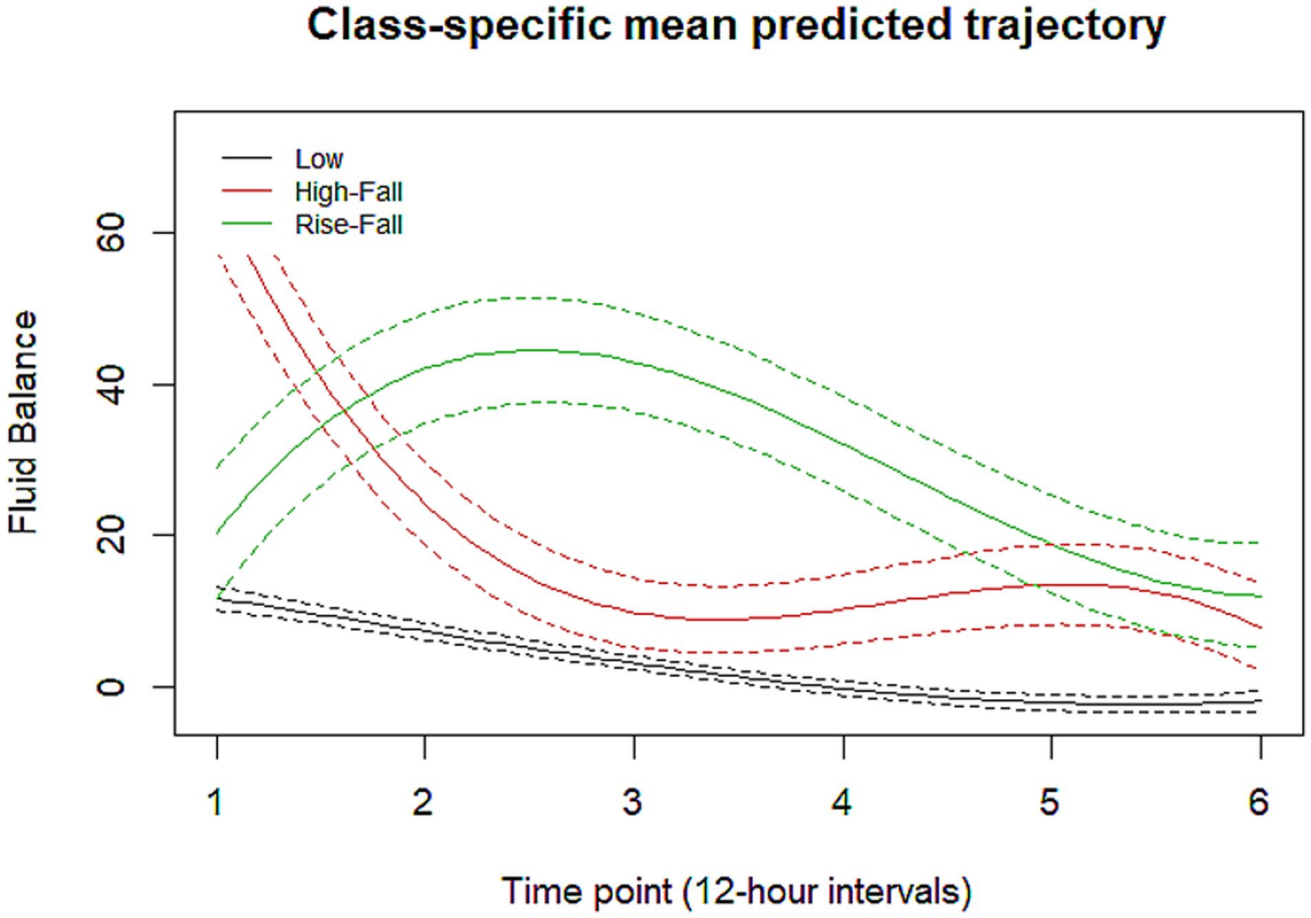
Fluid balance trajectories during the first 72 h after ICU admission in patients with acute pancreatitis in the MIMIC cohort.

Using LCMM on serial fluid balance measurements from the first 72 ICU hours, we identified three distinct trajectory classes (Figure 2):

Class 1: Low FB trajectory (*n* = 821, 80.02%): Characterized by a consistently low, near-neutral or mildly negative fluid balance throughout the observation period.
Class 2: High-Fall FB trajectory (*n* = 123, 11.99%): Marked by an initial phase of significant fluid overload (positive balance) within the first 12 h, followed by a rapid decline toward a negative balance over the subsequent 24 h.
Class 3: Rise-Fall FB trajectory (*n* = 82, 7.99%): Exhibited a progressive accumulation of fluid, peaking at approximately 48 h before gradually declining.

### Association between FB trajectories and in-hospital mortality

Associations between FB trajectories and in-hospital mortality are presented in Table 2. In the unadjusted model (Model 1), both the High-Fall FB (Class 2; HR = 2.731, 95% CI 1.784–4.179) and the Rise-Fall FB (Class 3; HR = 4.484, 95% CI 2.944–6.828) trajectories were associated with higher mortality than the Low FB trajectory reference (Class 1). Following adjustment for covariates selected via stepwise regression (Model 2), for Class 3, the elevated risk remained significant (HR = 2.286, 95% CI: 1.424–3.671), whereas the effect for Class 2 was no longer statistically significant (HR = 1.579, 95% CI 0.963–2.591). In a further multivariable model using hybrid Boruta feature selection+LASSO selection (Model 3), both trajectories showed independent associations with higher mortality risk: Class 2 (HR = 1.706, 95% CI: 1.036–2.811) and Class 3 (HR = 2.571, 95% CI: 1.576–4.196).

**Table 2:** Association between the fluid balance within 0–72 h and in-hospital mortality.

Variables	Model 1		Model 2		Model 3	
	HR (95% CI)	*p*	HR (95% CI)	*p*	HR (95% CI)	*p*
Fluid balance trajectories						
Low FB	Ref		Ref		Ref	
High-Fall FB	2.731 (1.784–4.179)	<0.001	1.579 (0.963–2.591)	0.07	1.706 (1.036–2.811)	0.036
Rise-Fall FB	4.484 (2.944, 6.828)	<0.001	2.286 (1.424–3.671)	<0.001	2.571 (1.576–4.196)	<0.001
Cumulative fluid balance						
CumFB	1.006 (1.005–1.007)	<0.001	1.003 (1.001–1.005)	0.002	1.003 (1.002–1.005)	<0.001
CumFB _class2	Ref		Ref		Ref	
CumFB _class1	0.686 (0.209–2.248)	0.534	0.494 (0.149–1.636)	0.248	0.485 (0.146–1.613)	0.238
CumFB _class3	3.011 (2.006–4.517)	<0.001	1.806 (1.154–2.826)	0.01	1.781 (1.137–2.790)	0.012

HR, hazard ratio; CI, confidence interval; Ref, reference; FB, fluid balance; CumFB, cumulative fluid balance. CumFB was categorized as follows: CumFB_class1 (CumFB ≤ − 50 ml/kg), CumFB_class2 (−50 ml/kg < CumFB < 25 ml/kg), and CumFB_class3 (CumFB ≥ 25 ml/kg). Model definitions: Model 1: univariable Cox regression. Model 2: multivariable Cox regression adjusted for age, gender, CKD, diuretic use, ventilation use, DBP, RDW, WBC, CCI and APS III. Model 3: multivariable Cox regression adjusted for age, gender, malignant cancer, diuretic use, heart rate, respiratory rate, RDW, TBil, WBC, Cr, CCI and APS III.

In parallel analyses, we performed multivariable linear regression on ICU LOS (Supplementary Table S4). Compared with the Low FB trajectory, the Rise–Fall FB trajectory was consistently associated with prolonged ICU LOS across all models (Model 1: *β* = 9.73, 95% CI 7.545–11.915; Model 2: *β* = 5.505, 95% CI 3.387–7.623; Model 3: *β* = 6.331, 95% CI 4.174–8.488). In contrast, the association with the High–Fall FB trajectory was markedly attenuated and no longer statistically significant after adjustment for confounders.

**Figure 3:**
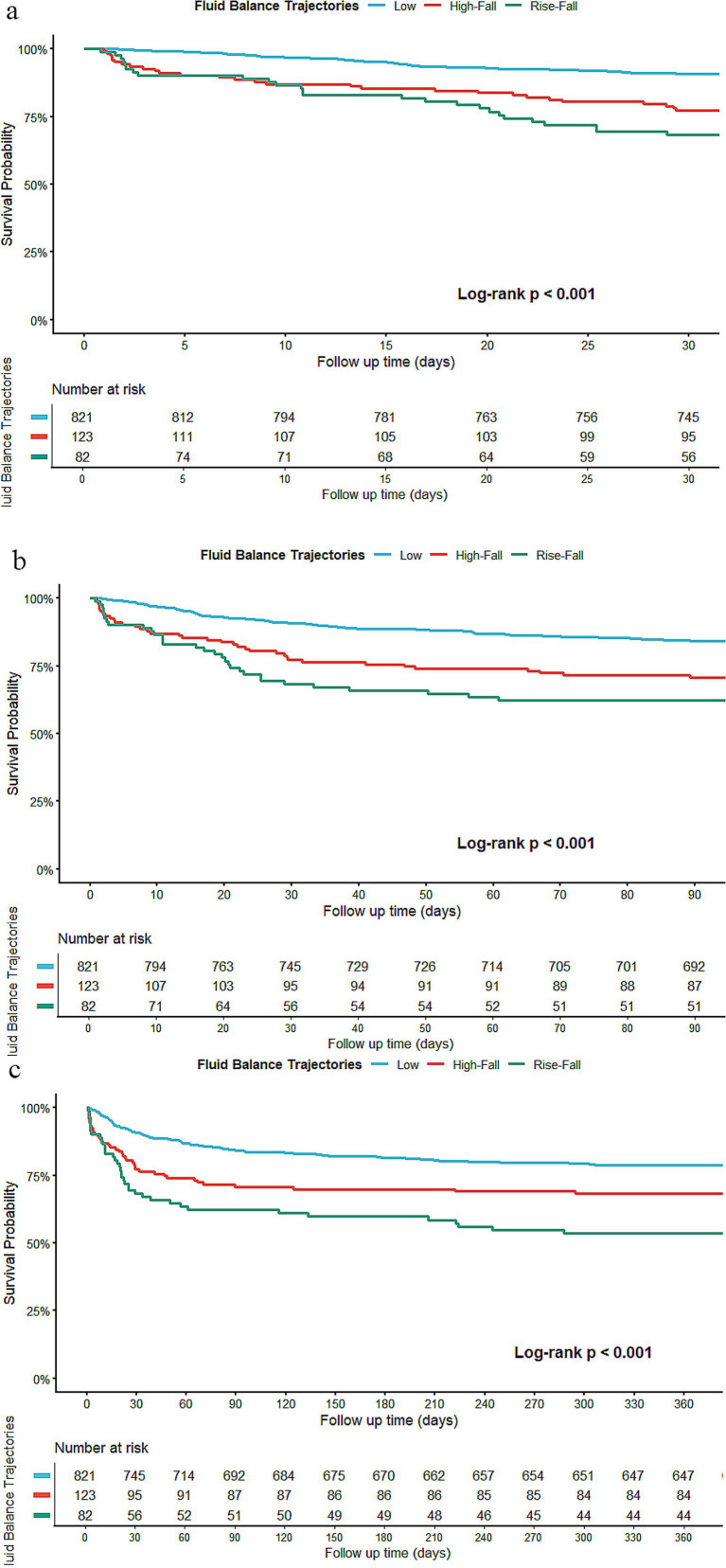
Kaplan–Meier curves for all-cause mortality stratified by fluid balance trajectories. Differences groups were assessed using log-rank test. **(A–C)** 30-day survival; **(B)** 90-day survival, and **(C)** 1-year survival.

Kaplan–Meier analysis demonstrated marked differences in survival across the FB trajectories at 30 days, 90 days and 1 year, with a stepwise decline in survival from Class 1 to Class 3 (log-rank *p* < 0.0001; Figure 3).

The primary finding for the Rise-Fall FB trajectory remained robust after IPTW analysis. After balancing baseline covariates—including demographic characteristics, laboratory parameters, comorbidities, and key severity scores (APS III, SAPS II, SOFA, and CCI)—Class 3 remained associated with higher mortality(HR = 2.708, 95% CI 1.454–5.042; Supplementary Table S5).

To further validate the robustness of these findings, we conducted two sensitivity analyses. First, excluding patients with an ICU length of stay less than 3 days (*n* = 471 excluded) did not materially alter the association between the Rise-Fall trajectory and mortality (Model 2: HR = 1.776, 95% CI 1.032–3.054; Model 3: HR = 2.034, 95% CI 1.153–3.585) (Supplementary Table S6). Second, in a competing-risks regression model treating early death as a competing event, the Rise-Fall trajectory remained strongly associated with an increased risk of post-72-h mortality (Model 2: HR = 2.405, 95% CI 1.368–4.230; Model 3: HR = 2.718, 95% CI 1.560–4.737) (Supplementary Table S7). These results confirm that our conclusions are robust to the exclusion of short-stay patients and are not unduly influenced by early mortality events.

Subgroup analyses were conducted to examine the consistency of the association between fluid balance trajectories and mortality across key clinical strata (Table 3). No statistically significant interactions were observed between FB trajectories and age, gender, mechanical ventilation, vasopressor use, or CKD status (all P for interaction >0.05). The Rise–Fall phenotype was consistently associated with increased mortality across most subgroups. However, certain strata with limited event counts (e.g., High-Fall FB in patients aged <60 years, both High-Fall FB and Rise-Fall FB in patients without vasopressor support, and CKD subgroups with ≤10 events) yielded wide confidence intervals and statistically unstable estimates. Therefore, all subgroup findings should be interpreted as exploratory.

**Table 3:** Subgroup analysis: Fluid balance trajectories and in-hospital mortality.

Subgroup	Variable	Events/N	HR_95 CI	*p*_value	*p*_for_interaction
Age<60 years	Low FB	29/428	1(Ref)		0.054
	High-Fall FB	6/72	0.79 (0.30–2.09)	0.637	
	Rise-Fall FB	13/51	2.06 (0.97–4.35)	0.059	
Age≥60 years	Low FB	50/393	1(Ref)		
	High-Fall FB	23/51	1.81 (1.00–3.27)	0.049	
	Rise-Fall FB	17/31	2.28 (1.22–4.26)	0.01	
Female	Low FB	30/326	1(Ref)		0.326
	High-Fall FB	15/60	1.9 (0.93–3.90)	0.078	
	Rise-Fall FB	17/37	2.74 (1.36–5.54)	0.005	
Male	Low FB	49/495	1(Ref)		
	High-Fall FB	14/63	1.12 (0.55–2.28)	0.752	
	Rise-Fall FB	13/45	2.00 (0.99–4.01)	0.052	
No-ventilation use	Low FB	49/581	1(Ref)		0.646
	High-Fall FB	14/66	1.43 (0.72–2.83)	0.304	
	Rise-Fall FB	12/33	1.79 (0.88–3.67)	0.11	
Ventilation use	Low FB	30/240	1(Ref)		
	High-Fall FB	15/57	1.41 (0.69–2.92)	0.347	
	Rise-Fall FB	18/49	2.51 (1.32–4.77)	0.005	
No-vasoactive use	Low FB	46/614	1(Ref)		0.767
	High-Fall FB	8/56	1.2 (0.51–2.81)	0.677	
	Rise-Fall FB	6/28	2.19 (0.82–5.89)	0.119	
Vasoactive use	Low FB	33/207	1(Ref)		
	High-Fall FB	21/67	1.81 (0.96–3.40)	0.067	
	Rise-Fall FB	24/54	2.69 (1.49–4.86)	0.001	
No-CKD	Low FB	58/674	1(Ref)		0.669
	High-Fall FB	21/101	1.30 (0.72–2.34)	0.381	
	Rise-Fall FB	21/64	2.03 (1.15–3.60)	0.015	
CKD	Low FB	21/147	1(Ref)		
	High-Fall FB	8/22	1.75 (0.59–5.23)	0.314	
	Rise-Fall FB	9/18	2.52 (0.89–7.12)	0.08	

HR, hazard ratio; CI, confidence interval; Ref, reference. Unless stated otherwise for specific subgroup analyses, multivariable Cox regression models were adjusted for age, sex, CKD, diuretic use, mechanical ventilation, DBP, RDW, WBC, CCI and APS III.

### Exploring dose–response between cumulative FB and mortality

**Figure 4:**
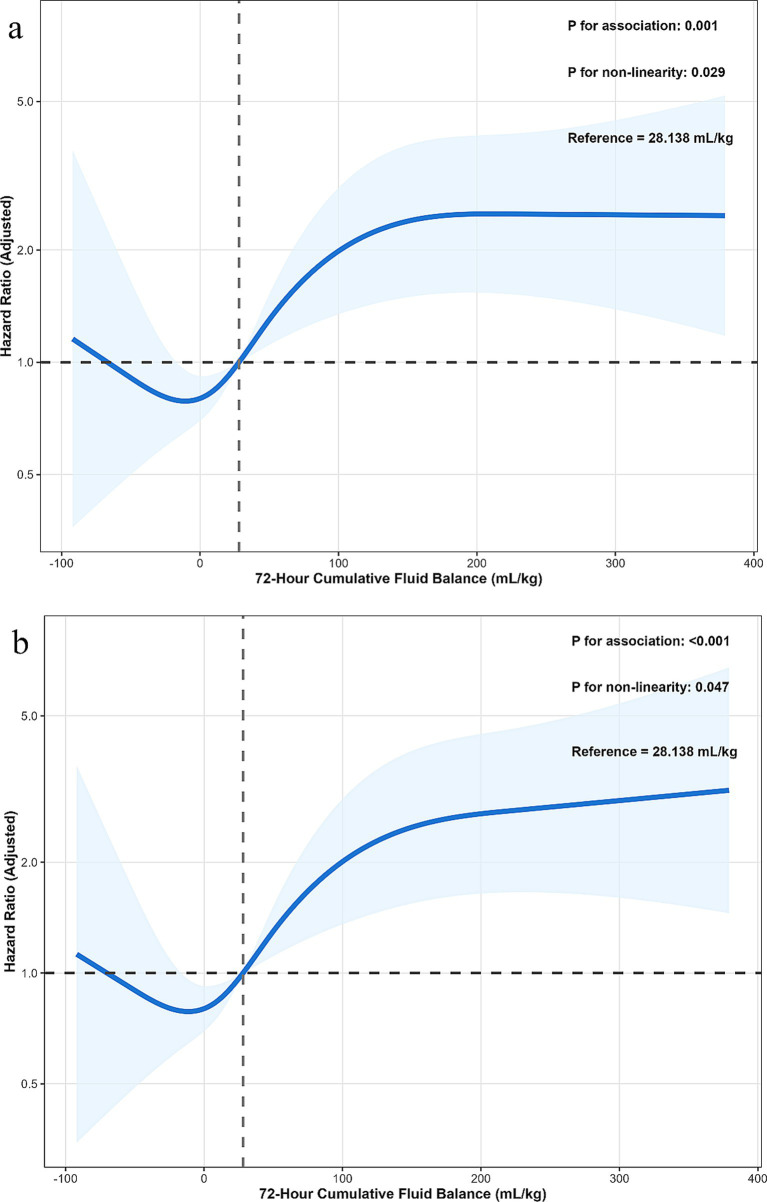
Dose–response relation between 0 and 72 h cumulative fluid balance and in-hospital mortality risk in the MIMIC cohort. The adjusted spline curves are shown. The inflection point occurs near 28.14 mL/kg. Results reflect the fully adjusted model (*p* < 0.05 for association and non-linearity effects). **(a)** Adjusted for age, gender, CKD, diuretic use, mechanical ventilation, DBP, CCI, RDW, WBC, and APS III. **(b)** Adjusted for age, gender, malignant cancer, diuretic use, heart rate, respiratory rate, RDW, TBil, WBC, Cr, CCI, and APS III.

When modeled as a continuous exposure, CumFB showed a statistically significant but small positive association with in-hospital mortality in Cox regression analyses (Model 1 HR = 1.006, 95% CI 1.005–1.007; Model 2 HR = 1.003, 95% CI 1.001–1.005; Model 3 HR = 1.003, 95% CI 1.002–1.005; Table 2). RCS analysis revealed a non-linear relationship between CumFB and mortality [(a) P for nonlinearity = 0.029; (b) P for nonlinearity = 0.047; Figure 4]. Guided by this curve, CumFB was categorized at −50 and 25 mL/kg: compared with the reference group (−50 to 25 mL/kg), CumFB > 25 mL/kg was independently associated with higher in-hospital mortality (Model 2 HR = 1.806, 95% CI 1.154–2.826; Model 3 HR = 1.781, 95% CI 1.137–2.790). Importantly, this heightened risk associated with CumFB > 25 mL/kg remained robust in a competing-risks regression model treating early death as a competing event (Model 2 SHR = 2.281, 95% CI 1.289–4.035; Model 3 SHR = 2.220, 95% CI 1.267–3.888; Supplementary Table S7) and was similarly mirrored by a significant prolongation of ICU length of stay in multivariable linear regression (Model 2 *β* = 1.538, 95% CI 0.300–2.777; Model 3 *β* = 1.781, 95% CI 1.137–2.790; Supplementary Table S4). In contrast, CumFB < −50 mL/kg showed no significant association with mortality.

### External validation in the eICU-CRD cohort

In the eICU-CRD cohort (*n* = 271; Supplementary Figure S1), we replicated the three dynamic FB trajectories identified in the MIMIC cohort—low FB, High-Fall FB and Rise-Fall FB (Supplementary Figure S2). As detailed in Supplementary Table S8, although the eICU cohort exhibited a higher CumFB (SMD = 0.233) and a higher severity of acute illness but a lower burden of chronic comorbidities, the overall distribution of FB trajectories was consistent between cohorts (SMD = 0.115), corroborating the cross-cohort stability of these phenotypes (Supplementary Table S8). Subsequent cox regression analyses (Supplementary Table S9) demonstrated that the Rise-Fall FB trajectory was independently associated with increased in-hospital mortality in univariate analysis (Model 1: HR = 8.627, 95% CI: 3.198–23.269) and in multivariable models adjusted for confounders (Model 2: HR = 6.862, 95% CI: 2.227–21.146; Model 3: HR = 9.583, 95% CI: 2.858–32.136).

Regarding CumFB, the nonlinear relationship with mortality could not be assessed in the eICU cohort due to the absence of outcome events in the patients with negative FB. Nevertheless, CumFB > 25 mL/kg remained an independent risk factor for mortality (Supplementary Table S9).

## Discussion

In this cohort of ICU-admitted AP patients, longitudinal modeling of early FB identified three discrete trajectory phenotypes that were differentially associated with in-hospital mortality. The Low FB trajectory was linked to the most favorable outcomes, while the High-Fall FB and Rise-Fall FB trajectories correlated with progressively worse prognosis. Notably, the Rise-Fall FB pattern—characterized by progressive accumulation peaking around 48 h followed by a delayed decline—consistently emerged as an independent marker associated with mortality across multivariable Cox, IPTW analyses and external validation. This robustness persisted after excluding short-stay patients and in competing-risks regression treating early death as a competing event. This underscores that early FB dynamics likely reflect the underlying severity of the disease course.

Previous studies mainly addressed static fluid metrics such as infusion rate and total volume (33, 34). Wan et al. further examined the prognostic relevance of dynamic infusion patterns (35). Extending this line of inquiry, we modeled FB as a time-varying series, integrating the dynamic interplay of fluid input and output to better capture the evolving pathophysiology of AP. In this context, the trajectories we identified correspond to distinct clinical phenotypes rather than mere mirrors of baseline severity. While the Low FB pattern is commonly associated with a milder inflammatory burden, it is associated with far more than just “mild disease.” Notably, even within this group, a considerable proportion required vasopressors (25.2%), mechanical ventilation (29.2%). This is consistent with the notion that maintaining a Low FB trajectory despite the need for organ support reflects a state of effective physiological compensation. The High-Fall profile is consistent with an initially critical presentation requiring aggressive resuscitation followed by successful active deresuscitation. Conversely, the Rise-Fall trajectory is characterized by a pathophysiological failure to transition from resuscitation to evacuation. Its reproducibility in an external cohort supports its identity as a unique biological phenotype, potentially corresponding to refractory capillary leak or persistent systemic inflammation. Crucially, the Rise-Fall trajectory remained independently associated with mortality after multivariable adjustment and IPTW. This suggests that FB trajectories provide prognostic information that extends beyond traditional severity quantification.

Timing is a critical dimension of fluid therapy. Interventions within the first 72 h after AP onset are widely regarded as decisive for outcome (36). Our finding that the Rise-Fall FB trajectory was associated with worse outcomes underscores the potential importance of early fluid management—particularly during the first 24 h after ICU admission. Failure to reverse early fluid accumulation or to transition promptly to evacuation within this window was likewise associated with a poorer prognosis (37). Similar timing-dependent effects have been reported in other critical conditions: studies in sepsis, ICU cardiac arrest and AMI with cardiogenic shock have shown increased mortality when fluid balance is not corrected within the second 24-h period or when neutral or negative balance is not achieved early (38–41).

Interestingly, the High-Fall group carried a lower mortality risk than the Rise-Fall pattern, possibly reflecting a rapid shift to active fluid removal or clinical improvement due to restoration of haemodynamic stability and vascular integrity. This observation aligns with the R. O. S. E. framework (resuscitation, optimization, stabilization, evacuation) for precision fluid management (13, 14). However, the observational design precludes causal inference: the trajectory may have influenced outcomes or simply reflected differences in underlying severity and treatment response.

Our subgroup analyses revealed no statistically significant interactions between fluid balance trajectories and key clinical covariates (age, gender, mechanical ventilation, vasopressor use, or CKD status) on mortality (all P for interaction >0.05). These findings indicate that the prognostic association of adverse fluid balance trajectories is broadly consistent across heterogeneous patient subgroups. However, the relevant subgroups were small and the interaction lacked statistical power; consequently, this observation should be considered exploratory and requires confirmation in sufficiently powered prospective studies.

In the MIMIC cohort, 0–72 h CumFB demonstrated a U-shaped association with in-hospital mortality. Markedly positive CumFB was independently associated with higher mortality, consistent with fluid overload–related tissue oedema and organ dysfunction, whereas marked negative balances showed no clear survival benefit and may reflect under-resuscitation or excessive fluid removal. External validation of this non-linear relationship in the eICU-CRD cohort was limited by a lack of events associated with negative fluid balance. Further analyses showed that a CumFB range of approximately −50 to 25 mL/kg over the first 72 h correlated with the better outcomes, while CumFB >25 mL/kg was independently associated with increased mortality. It is important to note that CumFB represents documented cumulative fluid exposure rather than actual anatomical volume, as unmeasured insensible losses and third-spacing may decouple recorded balances from true physiological status. These findings corroborate Huang X et al.’s report of a non-linear link between early FB and mortality in AP and align with observations in other critically ill populations that both extremes of FB are detrimental (23, 42, 43). Most importantly, these findings align with the prospective WATERFALL trial—a randomized controlled trial demonstrating that aggressive fluid resuscitation in acute pancreatitis increases the risk of fluid overload without improving clinical outcomes (9). Overall, the data are consistent with a relatively narrow therapeutic window after initial resuscitation and highlight the potential relevance of timely transition to active deresuscitation, which merits prospective evaluation.

The principal strength of this study is the use of LCMM on serial FB data to capture the temporal dynamics of fluid exposure and to delineate clinically meaningful phenotypes obscured by static metrics. Aid in identifying patients at high risk of persistent or progressive fluid accumulation, who might warrant closer monitoring of volume status and represent suitable candidates for evaluating timed fluid-removal strategies in future interventional studies. Thus, beyond serving as a quantitative risk-stratification tool, the framework provides a basis for future studies to refine the timing and targeting of fluid removal. Nonetheless, the optimal timing to initiate fluid removal remains to be defined by prospective research.

Several limitations warrant acknowledgment. First, the single-center, retrospective design limits causal inference and generalizability. Notably, the observed in-hospital mortality (13.5%) was lower than expected in high-income countries (e.g., 23% in a recent Dutch national ICU database (44)), potentially due to the exclusion of early deaths with missing records and other selection biases. Further validation in higher-acuity settings—particularly in low- and middle-income countries—is thus required. Second, residual confounding persisted due to insufficient data granularity. We could not precisely reconstruct pre-ICU resuscitation or vasopressor titration, nor differentiate fluid types (e.g., crystalloids vs. colloids). The lack of granular timestamps further precluded assessment of dynamic fluid management practices. Additionally, undocumented intake–output records inherent to retrospective analyses may have compromised data accuracy. Third, subgroup analyses were exploratory and potentially underpowered, necessitating future prospective validation.

## Conclusion

In patients with acute pancreatitis, the Rise–Fall FB trajectory, which reflects progressive fluid accumulation, was independently associated with in-hospital mortality. The nonlinear relationship between CumFB and mortality underscores the clinical imperative for balanced, time-sensitive fluid strategies that avoid sustained positive and extreme negative balances.

## Data Availability

Publicly available datasets were analyzed in this study. This data can be found at: The data supporting the findings of this study were derived from the following publicly available resources: Medical Information Mart for Intensive Care IV (MIMIC-IV) database, available at https://physionet.org/content/mimiciv/3.1/ eICU Collaborative Research Database (eICU-CRD), available at https://physionet.org/content/eicu-crd/2.0/ Access to these databases requires completion of a recognized training course in human subjects research and a data use agreement, as detailed on the PhysioNet website.
