# Supplementary material for: Association of fluid balance trajectories and cumulative fluid balance with in-hospital mortality in patients with acute pancreatitis
Source: Front Med (Lausanne). 2026 Jul 29;13:1863757. doi: 10.3389/fmed.2026.1863757 (PMC13461425; doi:10.3389/fmed.2026.1863757)
Supplement: Supplementary file 1 [file Supplementary_file_1.docx]

Supplementary Material

# Supplementary Tables:

Supplementary Table S1. LCMM parameters for 0-72-hour fluid balance trajectory grouping.

| Number of classes | Log likelihood | AIC | BIC | Entropy | Class 1 (%) | Class 2 (%) | Class 3 (%) | Class 4 (%) | Class 5 (%) |
| --- | --- | --- | --- | --- | --- | --- | --- | --- | --- |
| 1 | -23006.8 | 46029.54 | 46069.01 | 1 | 100 |  |  |  |  |
| 2 | -22752.8 | 45533.64 | 45602.71 | 0.622 | 81.87 | 18.13 |  |  |  |
| 3 | -22520.3 | 45080.53 | 45179.19 | 0.833 | 80.02 | 11.99 | 7.99 |  |  |
| 4 | -22436.7 | 44925.32 | 45053.58 | 0.823 | 1.07 | 75.54 | 15.5 | 7.89 |  |
| 5 | -22368.3 | 44800.62 | 44958.49 | 0.823 | 1.66 | 15.89 | 72.61 | 6.92 | 2.92 |

Note: AIC, Akaike Information Criterion; BIC, Bayesian Information Criteria.

Supplementary Table S2. Mean posterior probability under three classes of fluid balance trajectory.

| Class | Probability 1 | Probability 2 | Probability 3 |
| --- | --- | --- | --- |
| Class 1 | 0.946164002 | 0.034752624 | 0.019083374 |
| Class 2 | 0.109259387 | 0.871930669 | 0.018809944 |
| Class 3 | 0.092417266 | 0.0174616 | 0.890121133 |

Note:Class1: Low FB trajectory; Class2: High-Fall FB trajectory; Class3: Rise-Fall FB trajectory

Supplementary Table S3. Extended baseline characteristics according to fluid balance trajectories.

| Variables | Overall  (N=1026) | Low FB (n=821) | High-Fall FB (n=123) | Rise-Fall FB (n=82) | P |
| --- | --- | --- | --- | --- | --- |
| SpO2, % (median [IQR]) | 97 [94, 99] | 97 [94, 99] | 98 [94, 100] | 96 [94, 100] | 0.290 |
| SBP,mmHg (median [IQR]) | 125 [108, 144] | 128 [110, 145] | 118 [99, 132] | 122 [98, 143] | <0.001 |
| MBP,mmHg (median [IQR]) | 86 [73, 100] | 87 [75, 101] | 77 [68, 95] | 86 [71, 98] | 0.001 |
| BUN, mmol/L (median [IQR]) | 7.14 [4.28, 12.49] | 6.78 [3.93, 11.42] | 9.28 [5.36, 16.07] | 9.28 [5.80, 19.19] | <0.001 |
| Calcium, mmol/L (median [IQR]) | 2.00 [1.85, 2.12] | 2.02 [1.88, 2.15] | 1.85 [1.70, 1.99] | 1.96 [1.73, 2.09] | <0.001 |
| Sodium, mmol/L (median [IQR]) | 138.00 [135.00, 141.00] | 138.00 [135.00, 141.00] | 137.00 [133.00, 141.00] | 138.00 [134.00, 142.00] | 0.305 |
| Chloride, mmol/L (median [IQR]) | 104.00 [100.00, 109.00] | 104.00 [100.00, 109.00] | 106.00 [100.00, 111.00] | 103.00 [99.00, 107.75] | 0.058 |
| Potassium, mmol/L (median [IQR]) | 4.00 [3.60, 4.60] | 4.00 [3.60, 4.50] | 4.30 [3.80, 5.00] | 4.10 [3.70, 4.68] | 0.001 |
|  |  |  |  |  |  |
| Glucose, mmol/L (median [IQR]) | 7.27 [5.72, 9.77] | 7.05 [5.72, 9.55] | 7.77 [5.77, 11.27] | 7.55 [6.13, 10.50] | 0.040 |
| PT, s (median [IQR]) | 14.50 [13.00, 17.00] | 14.40 [12.90, 16.80] | 14.70 [13.15, 17.40] | 15.65 [13.05, 21.10] | 0.012 |
| ALP, U/L (median [IQR]) | 95.00 [67.00, 162.00] | 97.00 [69.00, 162.00] | 85.00 [58.00, 144.00] | 96.50 [65.25, 142.50] | 0.092 |
| AST, U/L (median [IQR]) | 69.00 [33.00, 172.75] | 63.00 [33.00, 146.00] | 80.00 [35.50, 210.00] | 104.00 [33.25, 244.00] | 0.017 |
| Congestive heart failure, n(%) | 202 (19.7) | 166 (20.2) | 17 (13.8) | 19 (23.2) | 0.178 |
| Chronic pulmonary disease, n(%) | 211 (20.6) | 170 (20.7) | 25 (20.3) | 16 (19.5) | 0.966 |
| Severe liver disease , n(%) | 113 (11.0) | 77 (9.4) | 22 (17.9) | 14 (17.1) | 0.004 |
| Diabetes, n(%) | 339 (33.0) | 272 (33.1) | 41 (33.3) | 26 (31.7) | 0.964 |
| Octreotide, n(%) | 45 (4.4) | 30 (3.7) | 4 (3.3) | 11 (13.4) | <0.001 |

Note: Data are presented as median [interquartile range] or number (percentage). IQR, Interquartile Range; SpO2: Oxyhemoglobin Saturation; SBP: Systolic Blood Pressure; MBP: Mean Blood Pressure; BUN: Blood Urea Nitrogen; PT: Prothrombin Time; ALP: Alkaline Phosphatase; AST: Aspartate Aminotransferase.

Supplementary Table S4. Association between the fluid balance within 0-72h and ICU length of stay.

| Variables | Model 1 | | Model 2 | | Model 3 | |
| --- | --- | --- | --- | --- | --- | --- |
|  | β(95%CI) | P | β(95%CI) | P | β(95%CI) | P |
| Fluid Balance Trajectories | | | | | | |
| Low FB | Ref |  | Ref |  | Ref |  |
| High-Fall FB | 3.955 (2.131-5.779) | <0.001 | 0.333 (-1.475-2.142) | 0.718 | 0.338 (-1.514-2.190) | 0.721 |
| Rise-Fall FB | 9.730 (7.545-11.915) | <0.001 | 5.505 (3.387-7.623) | <0.001 | 6.331 (4.174-8.488) | <0.001 |
|  | | | | | | |
| Cumulative Fluid Balance | | | | | | |
| CumFB | 0.043 (0.036-0.049) | <0.001 | 0.027 (0.020-0.035) | <0.001 | 0.030 (0.022-0.038) | <0.001 |
| CumFB  _class2 | Ref |  | Ref |  | Ref |  |
| CumFB  _class1 | 0.594 (-2.029-3.217) | 0.657 | -0.389 (-2.807-2.029) | 0.753 | -0.442 (-2.926-2.043) | 0.728 |
| CumFB  _class3 | 3.863 (2.611-5.114) | <0.001 | 1.538 (0.300-2.777) | 0.015 | 1.657 (0.393-2.920) | 0.010 |

Note: β, unstandardized regression coefficient;

Model definitions:

Model 1: univariable linear regression.

Model 2: multivariable linear regression adjusted for age, gender, CKD, diuretic use, ventilation use, DBP, RDW, WBC, CCI, and APS III.

Model 3: multivariable linear regression adjusted for age, gender, malignant cancer, diuretic use, heart rate, respiratory rate, RDW, TBil, WBC, Cr, CCI, and APS III.

Supplementary Table S5. Association between different fluid balance trajectories and in-hospital mortality: multiple inverse probability weighting methods.

| Method | Term | HR | HR_lower | HR_upper | p_value |
| --- | --- | --- | --- | --- | --- |
| Unweighted | class2 | 2.731 | 1.784 | 4.179 | <0.001 |
| Unweighted | class3 | 4.484 | 2.944 | 6.828 | <0.001 |
| IPTW | class2 | 2.383 | 1.291 | 4.400 | 0.006 |
| IPTW | class3 | 2.708 | 1.454 | 5.042 | 0.002 |
| entropy | class2 | 1.748 | 0.976 | 3.131 | 0.060 |
| entropy | class3 | 2.428 | 1.330 | 4.434 | 0.004 |
| overlap | class2 | 1.283 | 0.678 | 2.425 | 0.444 |
| overlap | class3 | 1.943 | 1.038 | 3.637 | 0.038 |
| treated | class2 | 3.037 | 1.484 | 6.215 | 0.0028 |
| treated | class3 | 2.802 | 1.198 | 6.549 | 0.017 |

Note:Class1: Low FB trajectory, as reference; Class2: High-Fall FB trajectory; Class3: Rise-Fall FB trajectory. IPTW: Inverse Probability of Treatment Weighting

Supplementary Table S6. Association between the fluid balance within 0-72h and in-hospital mortality after excluding patients with ICU length of stay < 72 hours.

| Variables | Model 1 | | Model 2 | | Model 3 | |
| --- | --- | --- | --- | --- | --- | --- |
|  | HR(95%CI) | P | HR(95%CI) | P | HR(95%CI) | P |
| Fluid Balance Trajectories | | | | | | |
| Low FB | Ref |  | Ref |  | Ref |  |
| High-Fall FB | 1.438 (0.848-2.441) | 0.1779 | 1.278 (0.704-2.318) | 0.420 | 1.395 (0.765-2.541) | 0.277 |
| Rise-Fall FB | 2.542 (1.556-4.154) | <0.001 | 1.776 (1.032-3.054) | 0.038 | 2.034 (1.153-3.585) | 0.014 |
|  | | | | | | |
| Cumulative Fluid Balance | | | | | | |
| CumFB | 1.004 (1.002-1.005) | <0.001 | 1.003 (1.001-1.005) | 0.010 | 1.004 (1.001-1.006) | 0.002 |
| CumFB  _class2 | Ref |  | Ref |  | Ref |  |
| CumFB  _class1 | 0.653 (0.194-2.197) | 0.491 | 0.432 (0.126-1.486) | 0.183 | 0.393 (0.113-1.374) | 0.143 |
| CumFB  _class3 | 2.205 (1.346-3.612) | 0.002 | 1.486 (0.852-2.594) | 0.163 | 1.500 (0.865-2.601) | 0.149 |

Model definitions:

Model 1: univariable Cox regression.

Model 2: multivariable Cox regression adjusted for age, gender, CKD, diuretic use, ventilation use, DBP, RDW, WBC, CCI and APS III.

Model 3: multivariable Cox regression adjusted for age, gender, malignant cancer, diuretic use, heart rate, respiratory rate, RDW, TBil, WBC, Cr, CCI and APS III.

Supplementary Table S7. Association between cumulative fluid balance within 0–72 hours and in-hospital mortality: a competing-risks analysis.

| Variables | Model 1 | | Model 2 | | Model 3 | |
| --- | --- | --- | --- | --- | --- | --- |
|  | SHR(95%CI) | P | SHR(95%CI) | P | SHR(95%CI) | P |
| Fluid Balance Trajectories | | | | | | |
| Low FB | Ref |  | Ref |  | Ref |  |
| High-Fall FB | 2.019 (1.175-3.467) | 0.011 | 1.377 (0.644-2.944) | 0.410 | 1.430 (0.678-3.013) | 0.350 |
| Rise-Fall FB | 4.148 (2.562-6.716) | <0.001 | 2.405 (1.368-4.230) | 0.002 | 2.718 (1.560-4.737) | <0.001 |
|  | | | | | | |
| Cumulative Fluid Balance | | | | | | |
| CumFB | 1.005 (1.004-1.007) | <0.001 | 1.004 (1.002-1.006) | 0.001 | 1.004 (1.002-1.007) | <0.001 |
| CumFB  _class2 | Ref |  | Ref |  | Ref |  |
| CumFB  _class1 | 1.043 (0.310-3.517) | 0.950 | 0.785 (0.253-2.441) | 0.680 | 0.742 (0.237-2.328) | 0.610 |
| CumFB  _class3 | 3.097 (1.892-5.07) | <0.001 | 2.281 (1.289-4.035) | 0.005 | 2.220 (1.267-3.888) | 0.005 |

Note: SHR, subdistribution hazard ratio; CI, confidence interval; Ref, reference; CumFB, cumulative fluid balance.

Model 1: Univariate Fine–Gray model.

Model 2: Multivariate Fine–Gray model adjusted for age, gender, CKD, diuretic use, ventilation use, DBP, RDW, WBC, CCI, and APS III.

Model 3: Multivariate Fine–Gray model adjusted for age, gender, malignant cancer, diuretic use, heart rate, respiratory rate, RDW, TBil, WBC, Cr, CCI, and APS III.

Supplementary Table S8. Comparison of baseline characteristics and fluid balance trajectories between the MIMIC derivation cohort and the eICU external validation cohort.

| Variables | MIMIC cohort | eICU cohort | SMD |
| --- | --- | --- | --- |
|  | N=1026 | N=271 |  |
| Trajectories (%) |  |  | 0.115 |
| Low FB | 821 (80.0) | 218 (80.4) |  |
| High-Fall FB | 123 (12.0) | 25 ( 9.2) |  |
| Rise-Fall FB | 82 ( 8.0) | 28 (10.3) |  |
| CumFB, mL/kg (mean (SD)) | 47.01 (87.21) | 87.28 (228.55) | 0.233 |
| Age (mean (SD)) | 58.29 (17.27) | 52.38 (16.61) | 0.349 |
| Gender = M (%) | 603 (58.8) | 160 (59.0) | 0.005 |
| Heart rate, beats/min (mean (SD)) | 101.54 (21.62) | 103.01 (22.73) | 0.067 |
| Respiratory rate, breaths/min (mean (SD)) | 21.69 (6.70) | 22.72 (7.11) | 0.149 |
| DBP,mmHg (mean (SD)) | 73.65 (19.42) | 74.45 (18.48) | 0.042 |
| Creatinine, umol/L (mean (SD)) | 147.63 (153.82) | 144.86 (145.86) | 0.019 |
| TBil, umol/L (mean (SD)) | 47.03 (87.21) | 30.78 (43.26) | 0.236 |
| WBC, ×10⁹/L (mean (SD)) | 14.17 (8.42) | 14.26 (7.20) | 0.012 |
| RDW, % (mean (SD)) | 15.28 (2.25) | 15.35 (2.65) | 0.029 |
| CKD = YES, n(%) | 187 (18.2) | 23 ( 8.5) | 0.289 |
| Malignant cancer = YES, n(%) | 78 ( 7.6) | 17 ( 6.3) | 0.052 |
| Diuretic use = YES, n(%) | 115 (11.2) | 104 (38.4) | 0.663 |
| Ventilation us = YES, n(%) | 346 (33.7) | 73 (26.9) | 0.148 |
| APS III (mean (SD)) | 53.58 (23.67) | 59.13 (27.24) | 0.217 |
| SOFA (mean (SD)) | 2.82 (2.94) | 5.82 (3.83) | 0.876 |
| SAPSII (mean (SD)) | 36.93 (16.22) | 31.79 (15.47) | 0.324 |
| CCI (mean (SD)) | 3.95 (2.94) | 2.24 (2.23) | 0.656 |

Supplementary Table S9. Association between the fluid balance and in-hospital mortality in the eICU-CRD cohort.

| Variables | Model 1 | | Model 2 | | Model 3 | |
| --- | --- | --- | --- | --- | --- | --- |
|  | HR(95%CI) | P | HR(95%CI) | P | HR(95%CI) | P |
| Fluid Balance Trajectories | | | | | | |
| Low FB | Ref |  | Ref |  | Ref |  |
| High-Fall FB | 6.033 (1.964-18.531) | 0.002 | 3.054 (0.889-10.493) | 0.076 | 4.1842 (1.222-14.333) | 0.023 |
| Rise-Fall FB | 8.627 (3.198-23.269) | <0.001 | 6.862 (2.227-21.146) | <0.001 | 9.583 (2.858-32.136) | <0.001 |
|  | | | | | | |
| Cumulative Fluid Balance | | | | | | |
| CumFB | 1.002 (1.001-1.003) | <0.001 | 1.004 (1.002-1.007) | <0.001 | 1.005 (1.003-1.008) | <0.001 |
| CumFB  _class2 | Ref |  | Ref |  | Ref |  |
| CumFB  _class1 | 0.000 (0.000-Inf) |  | 0.000 (0.000-Inf) |  | 0.000 (0.000-Inf) |  |
| CumFB  _class3 | 4.963 (1.154-21.339) | 0.031 | 5.036 (1.045-24.266) | 0.044 | 5.090 (1.079-24.022) | 0.040 |

Note:HR, hazard ratio; CI, confidence interval; Ref, reference; FB, fluid balance; CumFB, cumulative fluid balance.

CumFB was categorized as follows: CumFB_class1 (CumFB ≤ -50 ml/kg), CumFB_class2 (-50 ml/kg < CumFB < 25 ml/kg), and CumFB_class3 (CumFB ≥ 25 ml/kg).

Model definitions:

Model 1: univariable COX regression.

Model 2: multivariable COX regression adjusted for age, gender, CKD, diuretic use, ventilation use, DBP, RDW, WBC, CCI and APS III.

Model 3: multivariable COX regression adjusted for age, gender, malignant cancer, diuretic use, heart rate, respiratory rate, RDW, TBil, WBC, Cr, CCI and APS III.

## Supplementary Figures:

Supplementary Figure S1. Participant flow diagram illustrating inclusion and exclusion criteria for the eICU-CRD cohort.

Supplementary Figure S2. Fluid balance trajectories during the first 72 hours after ICU admission in patients with acute pancreatitis in the eICU-CRD cohort.
